# Modality-level attribution and redundancy-aware radiomics for MRI-based differentiation of melanoma and NSCLC brain metastases

**DOI:** 10.3389/fonc.2026.1816015

**Published:** 2026-06-29

**Authors:** Rafail C. Christodoulou, Georgios Vamvouras, Amal El Masri, Platon S. Papageorgiou, Evros Vassiliou, Elena E. Solomou, Sokratis G. Papageorgiou, Michalis F. Georgiou

**Affiliations:** 1 Division of Neuroimaging and Neurointervention, Department of Radiology, Stanford University, Stanford, CA, United States; 2 Department of Electrical and Computer Engineering, National Technical University of Athens (NTUA), Athens, Greece; 3 Faculty of Medicine, American University of Beirut, Beirut, Lebanon; 4 Department of Medicine, National and Kapodistrian University of Athens, Athens, Greece; 5 Department of Biological Sciences, Kean University, Union, NJ, United States; 6 Internal Medicine-Hematology, University of Patras Medical School, Rion, Greece; 7 1st Department of Neurology, Medical School, National and Kapodistrian University of Athens, Eginition Hospital, Athens, Greece; 8 Department of Radiology, University of Miami, Miami, FL, United States

**Keywords:** arteficial intelligence, explainable AI (SHAP), melanoma, NSCLC, personalized & precision medicine, radiomics

## Abstract

**Background:**

Differentiating the primary source of brain metastases using imaging alone is difficult, especially when intracranial disease is the initial sign of cancer. This study aimed to develop and validate a redundancy-aware, explainable radiomics framework for distinguishing melanoma from non–small cell lung cancer (NSCLC) brain metastases through multiparametric MRI.

**Methods:**

Lesion-level radiomic features were extracted from T1, contrast-enhanced T1 (T1CE), T2, and FLAIR sequences. Correlation-based redundancy filtering and L1-regularized feature ranking were applied to minimize collinearity among features. A structured parametric analysis across progressively larger top-K feature subsets was performed to evaluate performance stability and modality-specific contribution patterns. Multiple machine learning classifiers were tested using cross-validation and an independent test set, with model interpretability assessed via TreeSHAP to quantify feature- and modality-level contributions.

**Results:**

The best-performing configuration achieved a test AUC of 0.75, while RF-200 was selected for the primary tree-based explainability analysis due to its stable AUC-based discrimination. SHAP analysis showed that classification depended on a small set of predictors combining texture and intensity distribution features. FLAIR and T2 features contributed most to model attribution, suggesting that fluid-sensitive sequences capture discrimination-relevant heterogeneity and microenvironmental signals beyond contrast enhancement.

**Discussion:**

A redundancy-aware, interpretable radiomics approach shows promising preliminary discrimination between melanoma and NSCLC brain metastases and provides structured insight into modality-specific sequence contributions. External validation and prospective reader studies are required before clinical translation.

## Graphical Abstract

**Figure:**
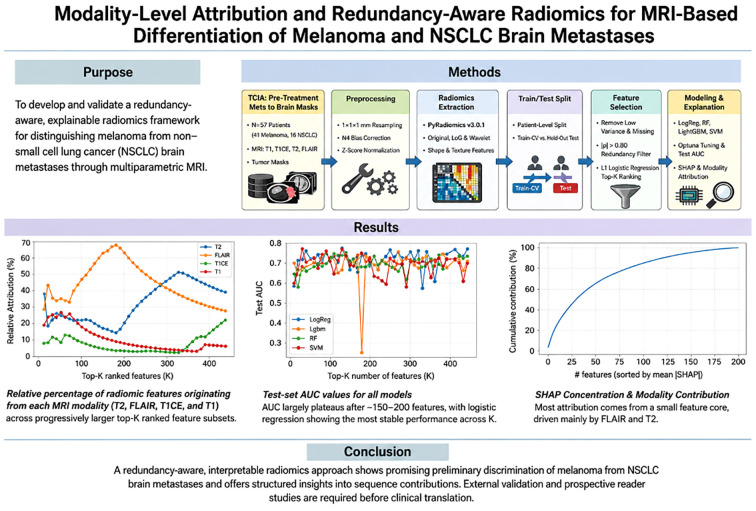

## Introduction

Brain metastases are the most frequent intracranial cancers in adults, causing significant neurologic morbidity and mortality related to cancer. Globally, lung cancer, breast cancer, and melanoma are responsible for most brain metastasis cases, with lung cancer being the primary tumor most commonly reported in many epidemiologic studies (1).

Lung cancer and melanoma are two common sources of brain metastases, with notable differences in their systemic treatment approaches. Lung cancer brain metastasis, particularly non–small cell lung cancer (NSCLC), accounts for about 50% of patients (2). NSCLC is biologically and therapeutically distinct from small-cell lung cancer, with distinct molecular targets and treatment strategies. NSCLC is the most prevalent histologic type, accounting for up to 85% of cases (3), and is often managed with targeted therapies and immunotherapy, as in melanoma (4, 5). Consequently, accurate differentiation between NSCLC and melanoma brain metastases is clinically meaningful and may directly influence treatment selection.

Radiomics is a computational technique that extracts high-dimensional quantitative features from medical images. These features are then used in machine learning (ML) models to develop predictive tools that analyze tumor phenotypic traits beyond visual inspection, thereby enabling predictions of tumor characteristics and biological behavior (6, 7). Radiomics has primarily been used to predict biomarkers, treatment responses, and overall survival in neuro-oncology (8). Only a few studies have investigated the possibility of determining the primary tumor source of brain metastases using radiomics. These research studies have demonstrated that MRI-derived radiomic models can differentiate metastases from lung cancer, breast cancer, and melanoma, with texture-based features often contributing substantially to classification performance (9–11).

Although previous research supports the overall feasibility of radiomic methods for predicting the primary tumor origin, several key methodological challenges remain. Many studies focus on multi-class classification across various primary sites, thereby weakening class-specific signals (11). Additionally, redundancy and collinearity among radiomic features are common in high-dimensional pipelines and can compromise model stability and interpretability if not properly managed. Moreover, while multiparametric MRI is frequently used in clinical settings, the specific contributions of individual modalities to classification accuracy have rarely been systematically quantified. In addition, most studies rarely employ explainability methods that not only provide insights into tumor characteristics but also enhance clinicians’ trust in the model’s predictions.

In this study, we developed an explainable, redundancy-aware, calibrated, and modality-specific radiomics framework to differentiate melanoma from NSCLC brain metastases. We extracted lesion-level radiomic features from four standard MRI sequences (T1, T1CE, T2, and FLAIR). To address collinearity and prevent information leakage, we applied correlation-based redundancy filtering combined with L1-regularized feature ranking. Several ML classifiers were tested through cross-validation and independent test sets. A structured parametric analysis across progressively larger top-K feature subsets was conducted to assess performance stability and quantify dynamic modality contributions. To improve interpretability, we included SHAP-based explainability and permutation importance analysis to quantify contributions at both the feature and modality levels. We hypothesized that a compact, high-performing subset of radiomic features would reliably distinguish melanoma from NSCLC metastases, and that specific MRI modalities would exhibit dominant attribution patterns, reflecting meaningful biological differences between tumor types.

## Methodology

### MRI data acquisition

Magnetic resonance imaging (MRI) data were obtained from the Pre-Treatment Mets to Brain Masks collection available on The Cancer Imaging Archive (TCIA) (12). This publicly available dataset contains pre-treatment brain MRI scans of patients with brain metastases, accompanied by expert-annotated tumor segmentation masks.

The dataset includes standard clinical brain MRI sequences acquired as part of routine diagnostic evaluation. Available modalities comprise contrast-enhanced T1-weighted (T1CE), T1-weighted (T1), T2-weighted (T2), and T2-FLAIR (FLAIR) images. Imaging was performed across multiple institution sites using both 1.5- and 3.0-T scanners from different vendors. Detailed acquisition parameters, including scanner manufacturer and sequence settings, are provided within the dataset metadata files on the TCIA website.

All imaging data were de-identified before public release in accordance with TCIA standards. The dataset provides corresponding tumor segmentation masks for each subject. According to the collection documentation, segmentation masks were generated during dataset curation and distributed alongside the imaging volumes.

### Ground truth and biological validation

The biological reference labels in this study were based on the documented primary tumor origin of each brain metastasis, classified as melanoma or NSCLC, as established through standard clinical and pathological assessment during routine patient care. Therefore, the model targets were clinically and biologically grounded disease labels rather than computationally inferred annotations.

### Code availability

The complete implementation of the preprocessing, radiomics extraction, feature selection, model training, calibration, and explainability pipeline is publicly available at: https://github.com/georgeDV2002/metastasis_radiomics.

### Descriptive statistics

Demographic data of the participants are shown in **Table 1**.

**Table 1:** Descriptive statistics.

Variable	Total (N = 57)	Melanoma (n=41)	NSCLC (n=16)	Test	p-value
Age, mean (SD)	63.1 (12.7)	62.9 (13.0)	63.6 (12.2)	Mann-Whitney U	0.894
Sex, n (%)					
Male	33 (57.9%)	25 (61.0%)	8 (50.0%)	χ2	0.6487
Female	24 (42.1%)	16 (39.0%)	8 (50.0%)		

### Radiomics extraction

For each patient in the dataset, the available MRI modalities included T1-weighted (T1), contrast-enhanced T1-weighted (T1CE), T2-weighted (T2), and T2-FLAIR (FLAIR) sequences. Radiomic features were extracted separately for each imaging modality and each tumor ROI. Literature suggests that lesion-level feature extraction preserves inter-lesion heterogeneity and avoids information loss introduced by ROI merging, thereby providing superior prognostic and predictive modeling performance (13, 14).

Prior to feature extraction, image volumes were resampled to isotropic 1 mm × 1 mm × 1 mm resolution to ensure spatial consistency across subjects. Bias-field inhomogeneity correction was applied using the N4 algorithm. Intensity normalization was performed via z-score normalization within the tumor mask. Radiomic feature extraction was implemented using PyRadiomics (v3.0.1), with the following standardized settings: bin width = 25, B-spline interpolation for image resampling, and nearest-neighbor interpolation for segmentation masks.

Features were extracted from the original image volumes, Laplacian-of-Gaussian (LoG) filtered images with σ = 0.5, 1.0, 2.0, and 3.0, and 3D wavelet-transformed images.

The resulting radiomic feature set encompassed multiple complementary feature families:

- Shape features, computed from the tumor mask on the original image, including descriptors such as volume, surface area, compactness, sphericity, elongation, and flatness.
- First-order statistics, describing the voxel intensity distribution within the tumor region, including mean, median, standard deviation, energy, entropy, skewness, kurtosis, and percentile-based metrics.
- Gray-Level Co-Occurrence Matrix (GLCM) features, quantifying spatial relationships between pairs of gray levels (e.g., contrast, correlation, homogeneity, angular second moment, entropy).
- Gray-Level Run-Length Matrix (GLRLM) features, characterizing consecutive runs of identical gray levels (e.g., short-run emphasis, long-run emphasis, gray-level non-uniformity, run-length non-uniformity).
- Gray-Level Size-Zone Matrix (GLSZM) features, measuring the size distribution of homogeneous gray-level zones (e.g., small-zone emphasis, large-zone emphasis, zone entropy, zone non-uniformity).
- Gray-Level Dependence Matrix (GLDM) features, assessing local gray-level dependencies (e.g., dependence entropy, dependence variance, low- and high-gray-level emphasis).
- Neighborhood Gray-Tone Difference Matrix (NGTDM) features, evaluating differences between a voxel and its neighborhood (e.g., coarseness, contrast, busyness, complexity, strength).

### Radiomic features importance ranking

The extracted radiomic features were divided into a training-cross-validation (train-CV) dataset and a held-out test dataset to ensure objective performance evaluation. To prevent data leakage at the patient level, the split was performed based on unique subject identifiers rather than individual lesions. Specifically, all lesions from a selected subset of patients were assigned exclusively to the test set, whereas the remaining patients formed the train-CV set.

The test set was constructed by randomly sampling a predefined number of patients from each class (melanoma and NSCLC), preserving the class representation ratio while ensuring that no patient appeared in both the train and test sets. The remaining patients constituted the train-CV dataset.

Feature importance was determined exclusively within the train-CV dataset to avoid information leakage from the test set. First, unsupervised filtering was applied, including removal of near-constant features (variance< 10^-8^), exclusion of features exceeding a maximum missingness threshold, and a Pearson correlation filter (|ρ| > 0.80) to reduce redundancy by removing highly correlated features. Feature ranking was then performed using an L1-regularized logistic regression model (solver = “saga”, class_weight = “balanced”) under stratified 2-fold cross-validation. Within each fold, median imputation and z-score standardization were fit on the training portion only, and model coefficients were extracted. Feature importance was quantified as the median absolute coefficient across folds that the logistic regression model assigned, and features were ranked accordingly. The top-K (K values tested) ranked features were retained and used to generate consistent training-CV and held-out test datasets for downstream modeling.

### Model development and hyperparameter optimization

Four machine learning model families were investigated: regularized logistic regression, Light Gradient Boosting Machine (LightGBM), random forest, and support vector machines (SVM). Within the train-CV dataset, stratified 2-fold cross-validation was employed to preserve class balance between melanoma and NSCLC samples across folds. Hyperparameter optimization was performed using Optuna, a Bayesian optimization framework that efficiently explores high-dimensional parameter spaces (15). Depending on the model family, the optimized parameters included the regularization strength and penalty type for logistic regression, the learning rate and tree-specific parameters for LightGBM, the ensemble size and depth for random forests, and the kernel and regularization parameters for SVM. The correlation threshold used during feature filtering was treated as a tunable hyperparameter, allowing the redundancy-reduction step to be optimized jointly with model training.

The objective function used during hyperparameter optimization was the mean area under the receiver operating characteristic curve (AUC) across cross-validation folds. AUC was selected as the primary metric due to its robustness to class imbalance and its independence from a fixed classification threshold. After identifying the optimal hyperparameter configuration within the train-CV set, each model was retrained on the full train-CV dataset using the selected parameters. Final evaluation was then performed on the independent held-out test set, which consisted of patients not included in the training stage. Performance was assessed using ROC-AUC and additional threshold-dependent metrics derived from model predictions. Given the class imbalance between melanoma and NSCLC, additional imbalance-aware and class-specific metrics were also computed, including PR-AUC, balanced accuracy, sensitivity, specificity, precision, and F1-score. Sensitivity refers to NSCLC detection, whereas specificity refers to melanoma classification. Threshold-dependent metrics on the held-out test set were computed using the Youden-derived threshold estimated from out-of-fold validation predictions.

### Explainability

To enhance the interpretability of the proposed radiomics models, multiple complementary explainability techniques were implemented, tailored to the underlying classifier architecture. For tree-based models (LightGBM and Random Forest), Shapley Additive Explanations (SHAP) were computed using the TreeSHAP algorithm. SHAP provides an additive decomposition of the model prediction for each sample:

$f\left(x\right)=\phi_{0}+\sum_{j=1}^{N_{f}}\phi_{j}$where *ϕ*_0_ represents the expected model output over the background distribution and *ϕ_j_* denotes the marginal contribution of the feature *j* to the prediction of the sample *x*. Positive SHAP values increase the probability of class 1 (NSCLC), whereas negative SHAP values decrease the probability of class 1 (NSCLC).

The following SHAP analyses were performed:

- Global feature importance based on mean absolute SHAP values.
- Pareto-style cumulative importance plots.
- Feature-level SHAP importance tables.
- Modality-level contribution analysis by aggregating SHAP values across features belonging to the same MRI modality.
- Average SHAP waterfall plots for correctly classified samples in each class.

For the Support Vector Classifier (SVC), model-agnostic KernelSHAP was employed. Given that SVC does not inherently provide probability estimates, calibrated probabilities (via Platt scaling fitted on out-of-fold predictions) were used to enable meaningful probabilistic explanations. KernelSHAP was applied on a bounded subset of test samples to maintain computational tractability.

In addition to SHAP-based interpretation, permutation importance was computed on the independent test set using AUC as the scoring metric. This procedure quantifies the decrease in discrimination performance when each feature is randomly permuted, thereby measuring the model’s reliance on that feature. Aggregating permutation importance across features enabled a secondary modality-level contribution analysis.

## Results

### Radiomic feature ranking and redundancy reduction

Feature importance was determined from the largest Top-K subset (K = 439), comprising all features that remained after strict ranking and correlation-based redundancy filtering. The retained feature pool spans all principal radiomic families, although with markedly unequal representation. Texture-based descriptors dominate the final subset, whereas purely morphological characteristics are nearly eliminated. Specifically, GLSZM (145/439) and First-order (102/439) features account for the largest fractions of the retained set, followed by GLCM (64/439), GLDM (57/439), GLRLM (35/439), and NGTDM (35/439). Shape descriptors are scarce after filtering, consistent with the expectation that geometric metrics exhibit high internal collinearity and limited incremental discriminative value when intensity- and texture-based descriptors are included.

**Figure 1:**
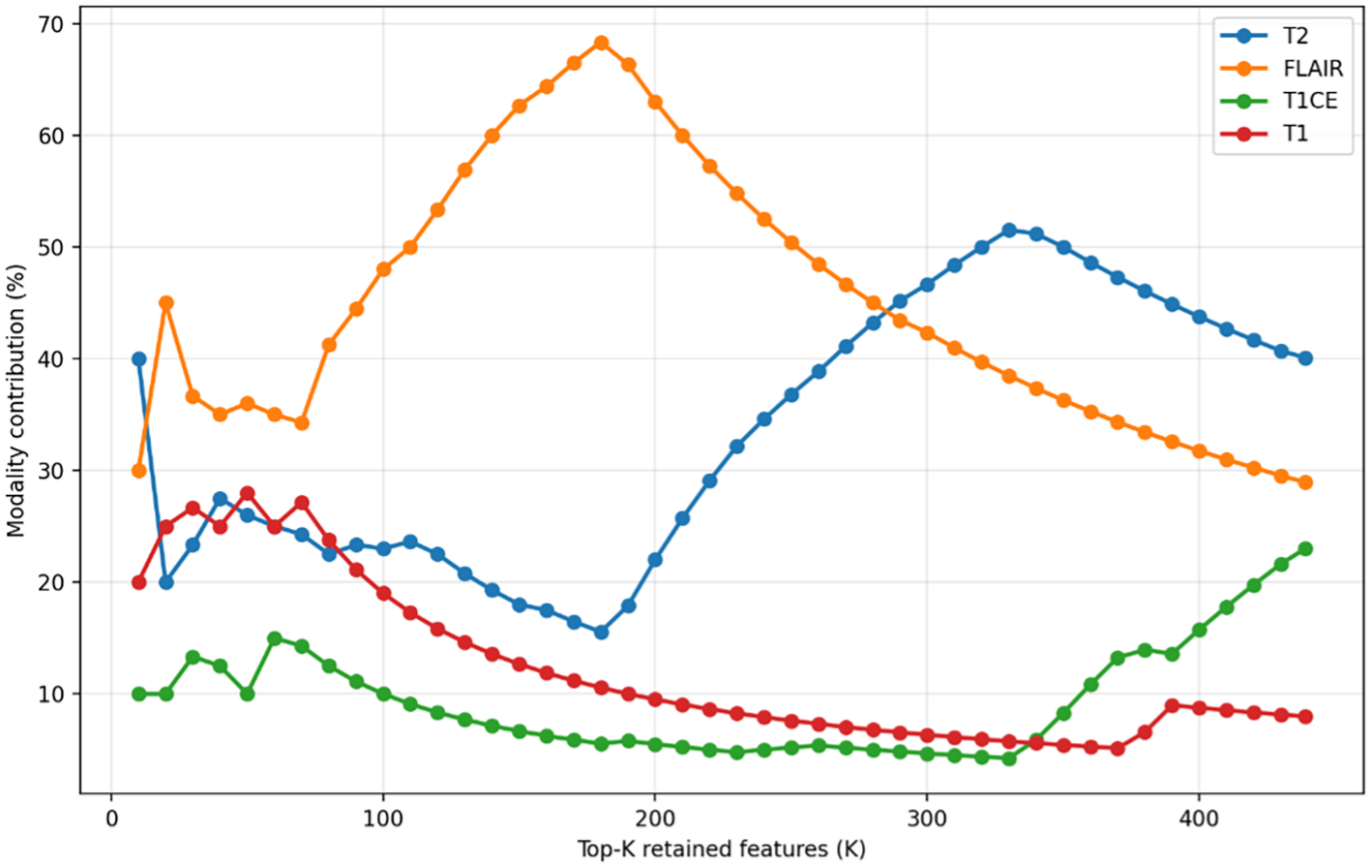
Relative percentage of radiomic features originating from each MRI modality (T2, FLAIR, T1-ce, and T1) across progressively larger top-K ranked feature subsets. At low K values, FLAIR-derived features dominate the highest-ranked positions. As K increases, FLAIR peaks and subsequently declines, while T2-derived features progressively increase and become predominant in larger subsets. T1 and T1-ce remain consistently underrepresented across all K values.

**Figure 1** depicts the relative percentage of features originating from each imaging modality (T2, FLAIR, T1-ce, and T1) as progressively larger subsets of top-ranked features are retained (K increasing in steps of 10). At very low K values, corresponding to the highest-ranked features, the distribution indicates that the most discriminative features are predominantly FLAIR-derived, with comparable but smaller contributions from T1 and T2, and minimal participation from contrast-enhanced T1-ce. As K increases, FLAIR rapidly strengthens its dominance, peaking at approximately 70% of the retained subset around K ≈ 180. Beyond this point, a steady shift is observed: FLAIR’s contribution progressively declines while T2 features increase correspondingly, gradually replacing FLAIR as the leading modality in the higher-K regime. Throughout the entire trajectory, both T1 and T1-ce remain consistently underrepresented relative to FLAIR and T2, exhibiting only modest fluctuations and confirming that the ranking procedure primarily prioritizes FLAIR-driven signal initially, followed by increasing incorporation of T2-derived descriptors at broader inclusion thresholds.

**Figure 2:**
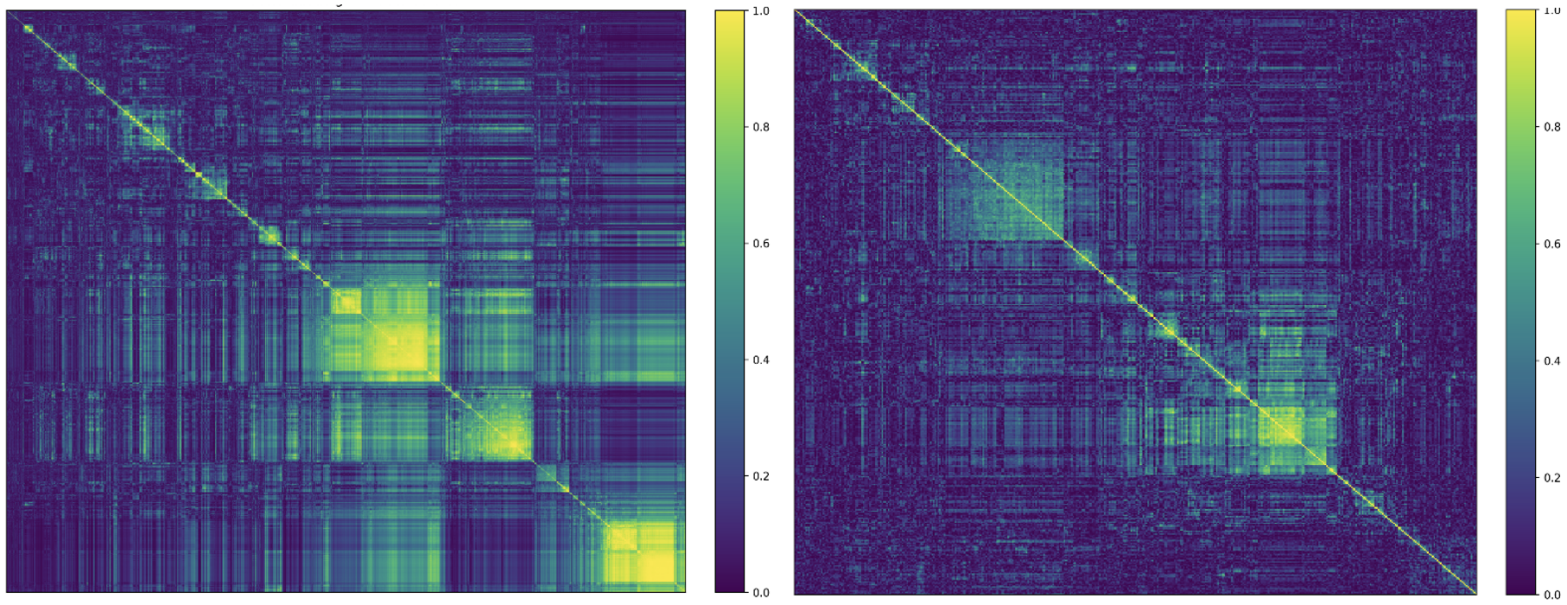
Absolute Pearson correlation matrices of the radiomic feature set prior to filtering (n = 4, 892 features) and after redundancy reduction (n = 439 features). Before filtering, dense high-correlation clusters (|r| ≈ 1) are evident, reflecting substantial inter-feature redundancy. After filtering, high-intensity blocks are markedly reduced, indicating effective attenuation of strong pairwise dependencies while preserving overall feature-space structure.

The impact of redundancy filtering on inter-feature dependency is illustrated in **Figure 2**, which presents the absolute Pearson correlation matrices before (n = 4892) and after (n = 439) filtering. Prior to filtering, the matrix exhibits extensive high-intensity blocks along and around the diagonal, reflecting clusters of highly correlated features (|r| approaching unity). These blocks are characteristic of radiomic pipelines in which multiple filters and parametrizations generate families of descriptors that capture overlapping spatial information. After filtering, the matrix becomes substantially darker overall, with a marked contraction of bright clusters. The reduction in high-intensity regions indicates effective attenuation of strong pairwise dependencies while maintaining the global structural organization of the feature space.

**Figure 3:**
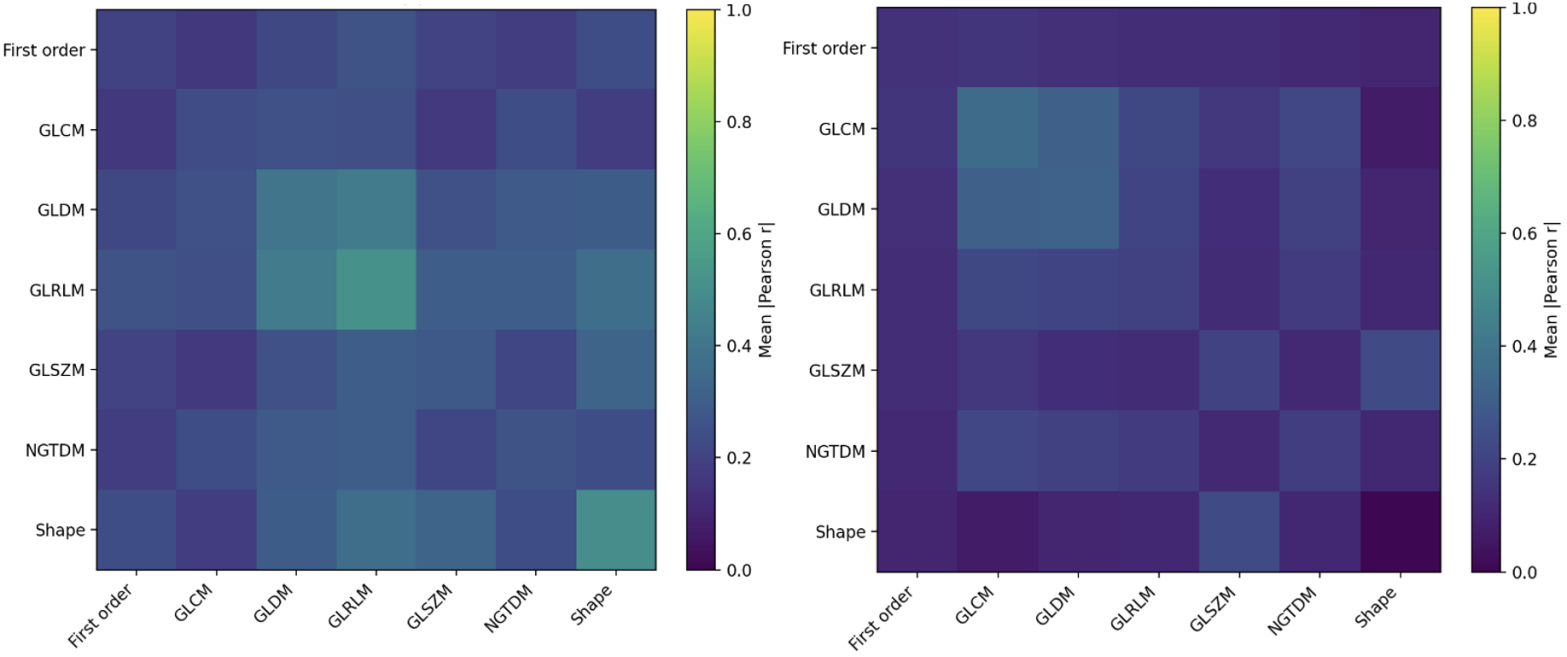
Heatmaps illustrating mean absolute Pearson correlation values within and between radiomic feature families before and after redundancy filtering. Intra-family correlations are substantially reduced following filtering, particularly within texture-based families (e.g., GLDM, GLRLM), demonstrating cluster-level redundancy attenuation. Cross-family correlations are also diminished, indicating improved independence across descriptor groups.

Family-level mean correlations further clarify these structural changes. As shown in **Figure 3**, intra-family correlations are generally elevated before filtering, particularly within GLDM and GLRLM groups, where dense internal redundancy is evident. Cross-family correlations are also observable among texture families, suggesting shared second-order spatial dependencies. Following filtering, mean |r| values decrease across nearly all family blocks. Intra-family blocks exhibit darker coloration, and cross-family interactions are attenuated, indicating that redundancy reduction operates not only at the level of individual feature pairs but also at the level of correlated descriptor clusters within and between radiomic families.

**Figure 4:**
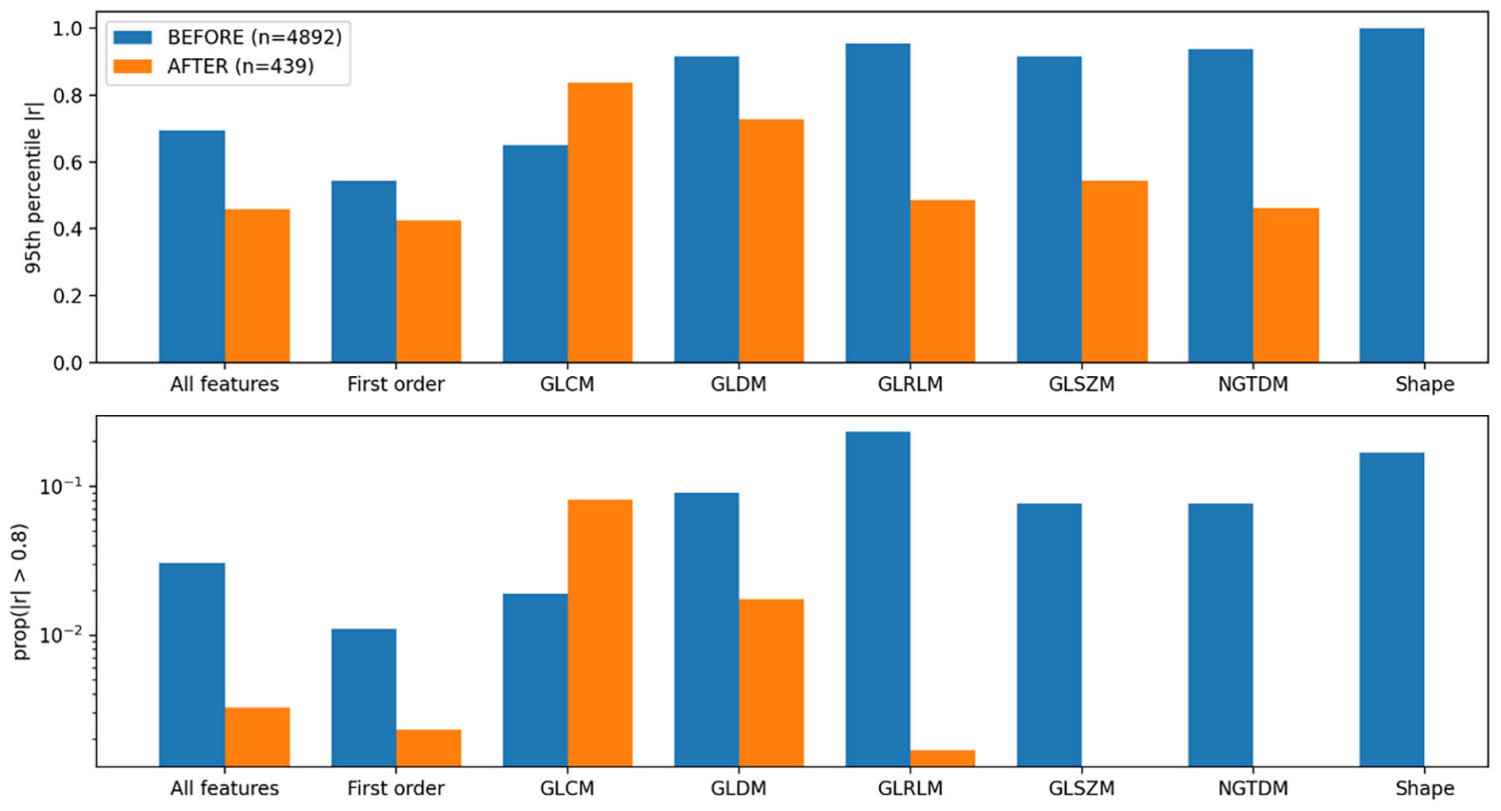
Upper-tail correlation metrics across radiomic feature families. Left panel: 95th percentile of absolute Pearson correlation (|r|). Right panel: proportion of feature pairs exceeding a high-correlation threshold (|r| > 0.8). Redundancy filtering reduces extreme pairwise dependency across most families, with particularly pronounced effects in texture-based descriptors.

To quantify changes in the upper tail of the correlation distribution, the 95th percentile of |r| and the proportion of feature pairs exceeding a high-correlation threshold (|r| > 0.8) were evaluated, as shown in **Figure 4**. Across the overall feature set and most individual families, the 95th percentile decreases after filtering, indicating reduced extreme pairwise dependency. The attenuation is particularly evident in texture families such as GLCM, GLDM, and GLSZM. More notably, the proportion of strongly correlated pairs declines substantially, in some families by approximately an order of magnitude, demonstrating that the filtering procedure preferentially removes near-duplicate descriptors while preserving moderately correlated features that may encode complementary information.

**Figure 5:**
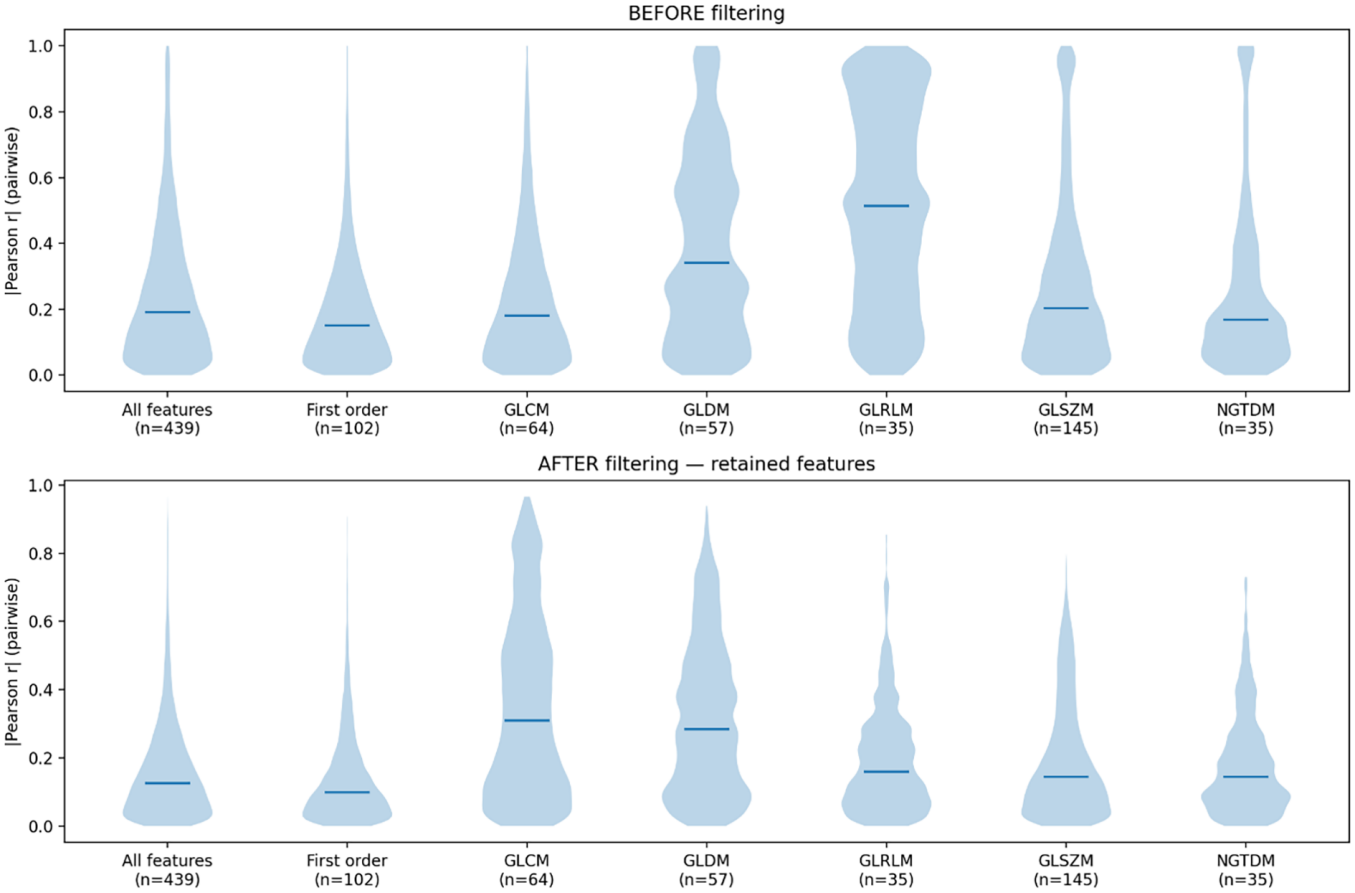
Violin plots depicting the distribution of pairwise absolute Pearson correlations (|r|) within each radiomic feature family before and after redundancy filtering. Following filtering, distributions exhibit reduced spread, lower median |r| values, and shortened upper tails, reflecting a globally less collinear feature space.

The distributional behavior of pairwise correlations is illustrated in **Figure 5**. Before filtering, several families display broad distributions with pronounced upper tails extending toward |r| ≈ 1.0, consistent with dense clusters of redundant descriptors. After filtering, the distributions become visibly narrower, with reduced spread and shortened upper tails. Median |r| values decrease in most families, reflecting improved independence among retained descriptors. The contraction of these distributions confirms that redundancy reduction is not limited to extreme outliers but results in a globally less collinear feature space.

### Classification

To systematically evaluate the influence of feature subset size on model performance, a structured top-K analysis was conducted, in which progressively larger subsets of the ranked feature list were retained and evaluated independently. For each value of K∈{10, 20, 30, 40, 60,…, 439}, the pipeline independently performed ranking, dataset splitting, and model training.

**Figure 6:**
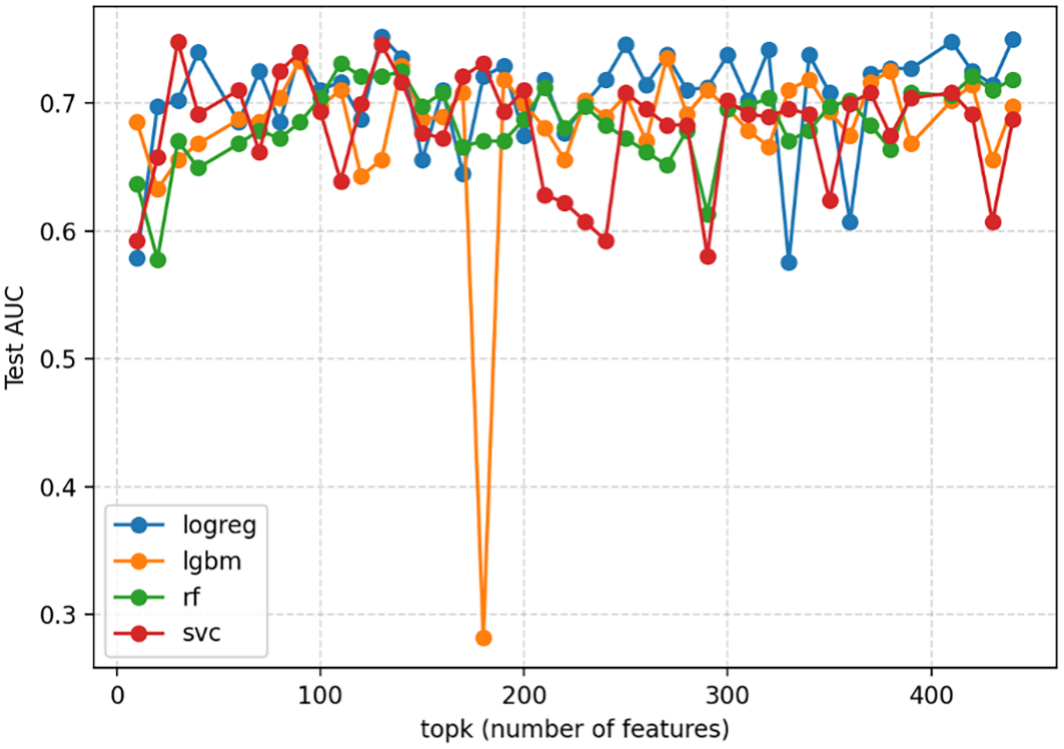
Independent test-set area under the receiver operating characteristic curve (AUC) for logistic regression, LightGBM, random forest, and SVC models across progressively larger top-K feature subsets. Logistic regression demonstrates the most stable performance across K values. Performance plateaus beyond moderate K, indicating diminishing returns from inclusion of additional lower-ranked features.

**Figure 6** summarizes the resulting test-set AUC values for all models as a function of K. Logistic regression exhibits the most consistent performance across the evaluated range, maintaining AUC values predominantly between 0.70 and 0.75, with limited variation. LightGBM achieves competitive performance for moderate-to-large K values but exhibits greater variability, including a marked decline at *K* ≈ 180. Random forest shows comparatively smoother behavior but slightly lower average performance, generally clustering between 0.66 and 0.72 depending on K. The SVC model exhibits moderate variability with occasional local peaks yet is overall less stable than logistic regression.

Performance does not increase monotonically with K. Instead, AUC values plateau beyond approximately *K* ≈ 150–200, indicating that the highest-ranked subset captures the majority of the predictive signal. Inclusion of additional features yields diminishing performance gains and, in certain cases, introduces additional variance. These findings are consistent with the preceding redundancy-filtering strategy, suggesting that compact, high-ranking feature subsets achieve comparable or improved generalization relative to larger feature pools.

**Figure 7:**
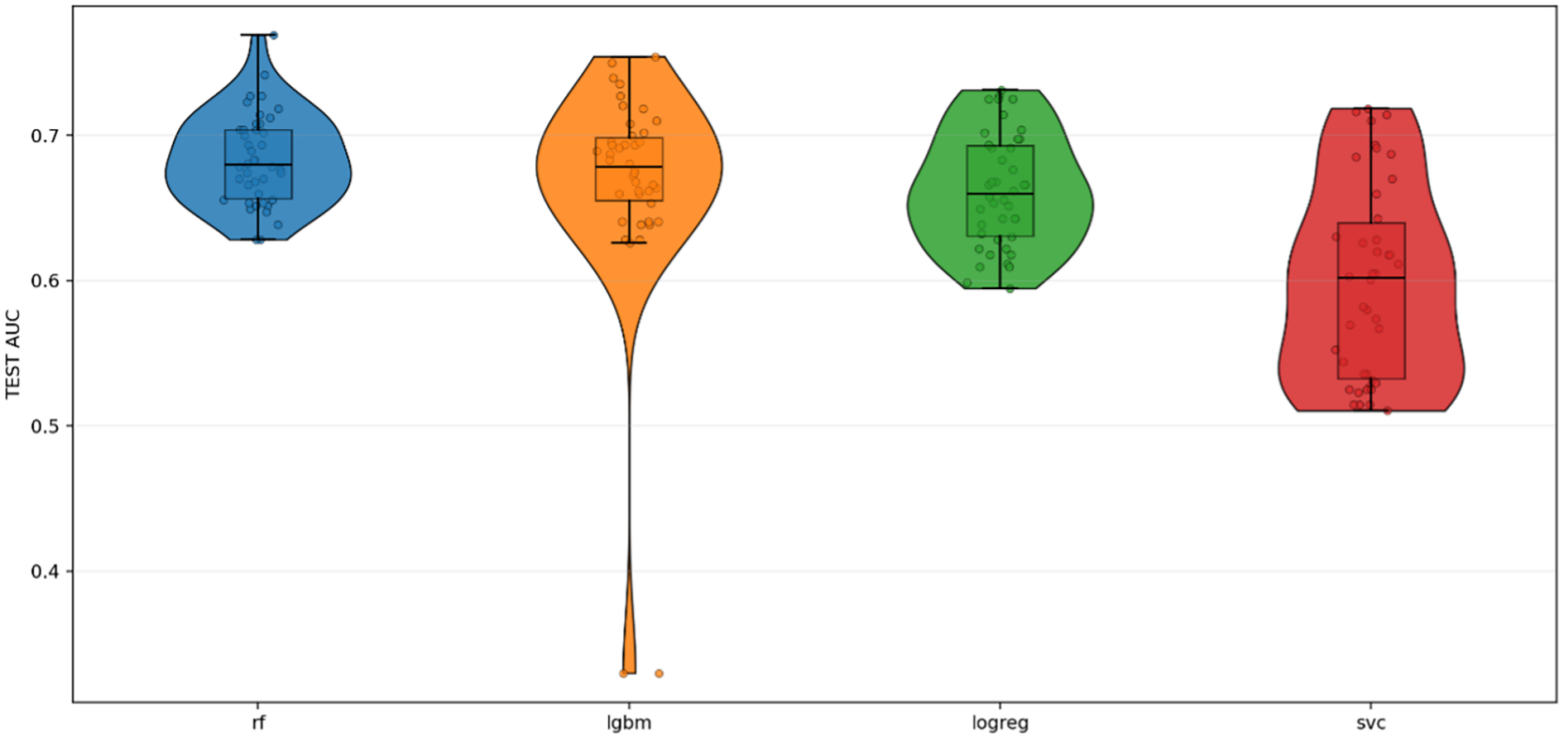
Distribution of independent test-set AUC values for each model family across all evaluated top-K configurations. Logistic regression and random forest exhibit more compact distributions and higher central tendency, indicating greater stability. LightGBM and SVC show increased dispersion and variability.

To further assess model robustness, **Figure 7** illustrates the distribution of test-set AUC values across all evaluated K values for each model family. Logistic regression and random forest show relatively compact distributions with higher median AUC values, indicating stable performance across feature subset sizes. LightGBM presents greater dispersion, including isolated low-performance outliers. SVC exhibits the greatest variability and the lowest central tendency. These results support the observation that logistic regression provides the most consistent generalization behavior within this framework.

Among the configurations evaluated, three representative models were selected for detailed reporting because they showed complementary performance profiles across discrimination and class-specific metrics. RF-200 retained strong AUC-based discrimination on the held-out test set (AUC = 0.74), but its threshold-dependent NSCLC sensitivity was limited (sensitivity = 0.29; balanced accuracy = 0.55), indicating that its probability ranking was stronger than its fixed-threshold classification behavior. LGBM-220 provided the most balanced test-set profile, with AUC = 0.75, PR-AUC = 0.82, accuracy = 0.69, balanced accuracy = 0.72, sensitivity = 0.61, specificity = 0.82, and F1-score = 0.71. SVC-100 achieved the highest NSCLC sensitivity and F1-score on the test set (sensitivity = 0.75; F1-score = 0.74), although this was accompanied by lower specificity (0.53) (**Table 2**). These findings indicate that model ranking depends on whether discrimination performance or threshold-dependent class-specific performance is prioritized, and that imbalance-aware metrics provide a more complete assessment than accuracy or ROC-AUC alone.

**Table 2:** Validation and independent test-set classification metrics for the three selected model configurations.

Metric	RF | top k = 200		LGBM | top k = 220		SVC | top k = 100	
	OOF validation	Test	OOF validation	Test	OOF validation	Test
AUC	0.76	0.74	0.78	0.75	0.79	0.71
PR-AUC	0.26	0.80	0.30	0.82	0.46	0.82
Accuracy	0.71	0.49	0.60	0.69	0.64	0.67
Balanced accuracy	0.80	0.55	0.77	0.72	0.76	0.64
Sensitivity	0.93	0.29	1.00	0.61	0.93	0.75
Specificity	0.67	0.82	0.53	0.82	0.59	0.53
Precision	0.33	0.73	0.27	0.85	0.29	0.72
F1-score	0.49	0.41	0.43	0.71	0.44	0.74

OOF validation metrics were computed from out-of-fold predictions, while test-set threshold-dependent metrics were computed using the OOF Youden-derived threshold. Sensitivity refers to NSCLC detection, whereas specificity refers to melanoma classification.

### Explainability

**Figure 8:**
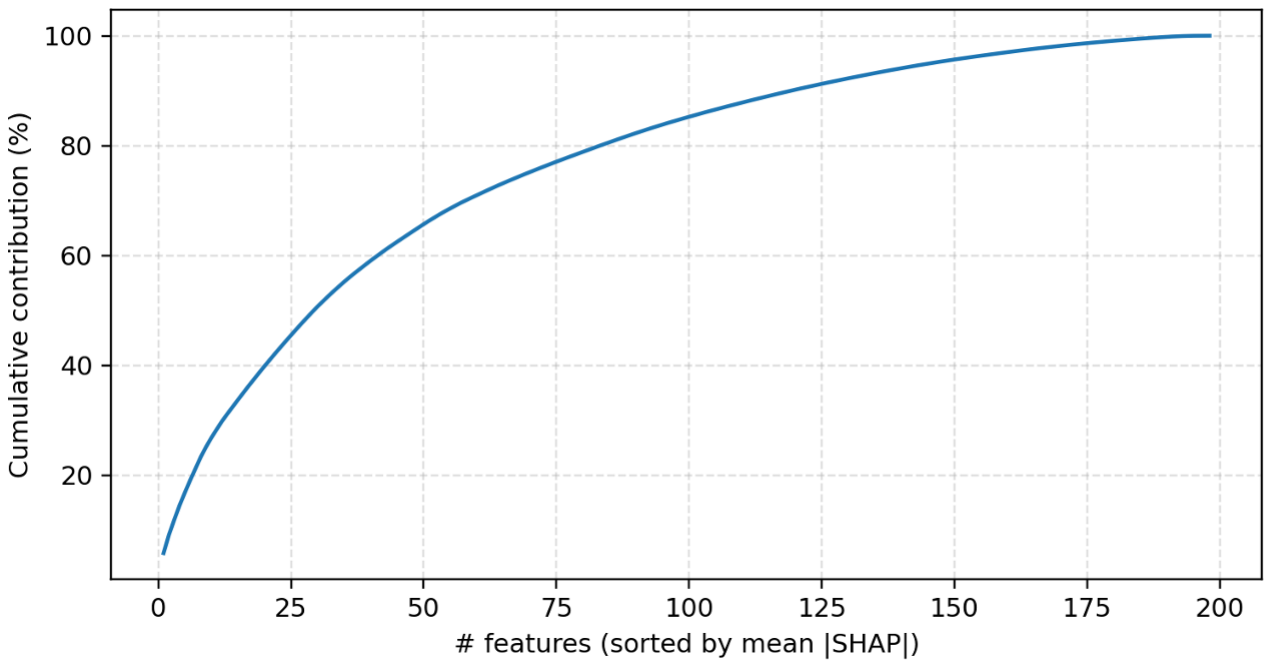
Cumulative contribution curve of radiomic features ranked by mean absolute SHAP value on the independent test set. The rapid initial increase followed by gradual saturation indicates that a limited subset of predictors accounts for the majority of the model’s attribution mass.

The TreeSHAP algorithm was applied to RF-200, the selected tree-based model used for the primary explainability analysis, on the independent test set, and mean absolute SHAP values were aggregated at both the feature and modality levels. When features were ranked by mean absolute SHAP value, the cumulative importance curve (**Figure 8**) demonstrated a rapid increase followed by progressive saturation, indicating that a limited subset of predictors accounts for the majority of the model’s attribution mass. This behavior supports the presence of a dominant core of radiomic features that drives the classification decision, with additional features contributing only marginal explanatory gain.

At the modality level, aggregation of SHAP values revealed a clear hierarchy of contribution. FLAIR-derived features accounted for 49.5% of the total mean absolute SHAP attribution, followed by T2 (31.1%), T1 (11.5%), and T1ce (7.9%). These proportions indicate that the model’s decision function is primarily driven by FLAIR and T2 radiomic descriptors, while T1 and T1ce provide comparatively smaller but non-negligible contributions. Importantly, this distribution reflects the relative explanatory weight the model assigns to unseen data, rather than feature frequency alone, thereby providing a quantitative characterization of modality-specific influence within the predictive framework.

**Figure 9:**
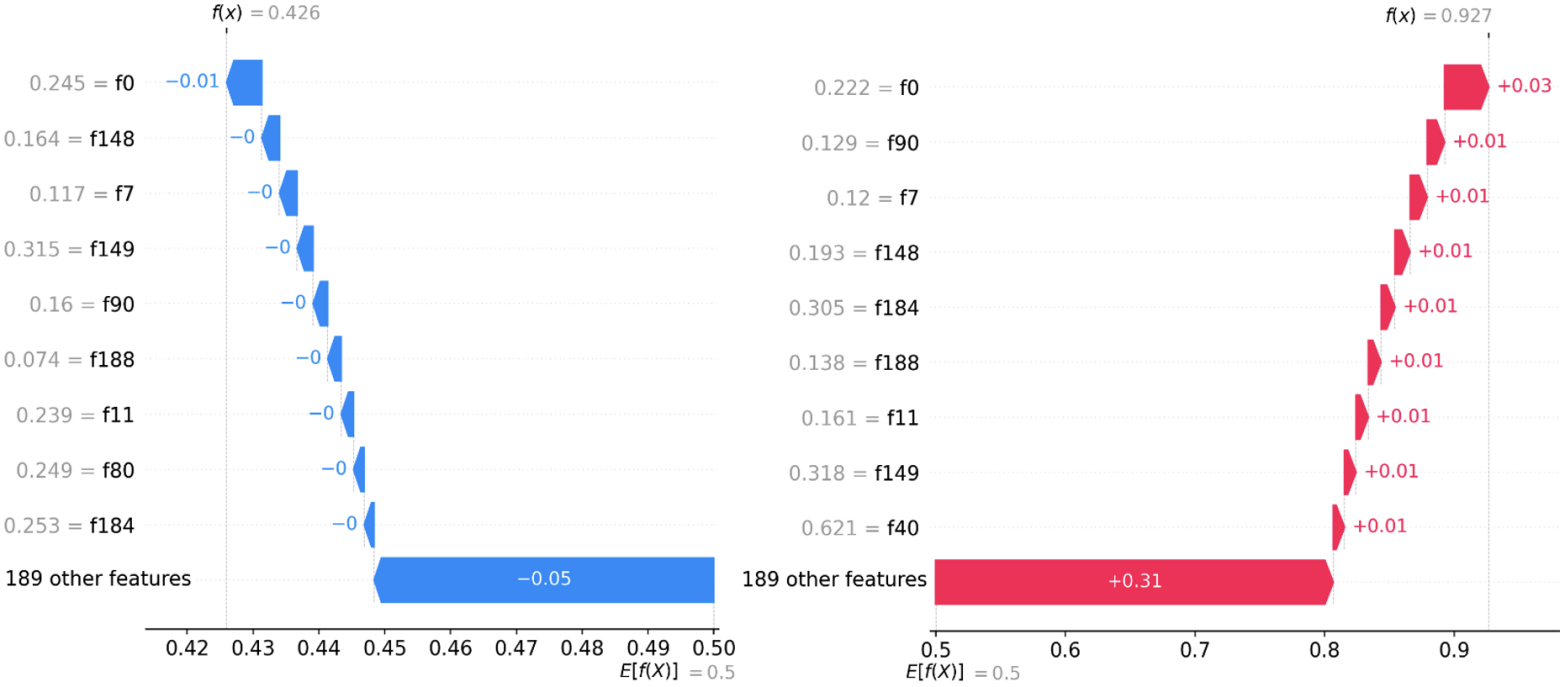
Waterfall diagrams for representative correctly classified class-0 (left) and class-1 (right) test samples. Each bar represents the contribution of an individual feature to the final model output f(x), starting from the baseline expectation E[f(X)]. Red bars indicate contributions toward the positive class, while blue bars indicate contributions toward the negative class. For visualization clarity, only the top contributing features are shown, with the remaining features aggregated.

**Figure 9** presents SHAP waterfall diagrams for representative correctly classified class-0 (left) and class-1 (right) samples. The length and color of each bar denote the magnitude and direction of the feature’s contribution to the prediction, with red indicating influence toward the positive class and blue toward the negative class. Starting from the baseline expectation E[f(X)], individual feature contributions are algebraically summed to obtain the final model output f(x). For clarity, only the most influential predictors are displayed, while the remaining features are aggregated. Their full radiomic descriptions are provided in **Table 3**.

**Table 3:** Radiomic feature index and details.

Index	Modality	Filter	Family	Feature
0	T2	Wavelet HLH	GLSZM	GrayLevelVariance
7	T1	Wavelet HLH	NGTDM	Contrast
11	T1	Wavelet LLL	GLSZM	GrayLevelVariance
40	T1	Log σ=0.5mm	GLSZM	GrayLevelVariance
80	T2	Log σ=0.5mm	GLSZM	LowGrayLevelZoneEmphasis
90	FLAIR	Wavelet LLL	FIRSTORDER	Maximum
148	FLAIR	Wavelet HLH	FIRSTORDER	Kurtosis
149	FLAIR	Wavelet HLH	FIRSTORDER	Skewness
184	T2	Wavelet HLH	GLSZM	LowGrayLevelZoneEmphasis
188	T2	Wavelet HHL	FIRSTORDER	Mean

## Discussion

Our redundancy-aware, modality-specific radiomics framework showed preliminary ability to differentiate melanoma from NSCLC brain metastases in an internally evaluated cohort. Performance was not uniform across all metrics: RF-200 retained strong AUC-based discrimination, LGBM-220 showed the most balanced threshold-dependent test-set profile, and SVC-100 favored NSCLC sensitivity. Importantly, model attribution analysis revealed that a limited subset of radiomic features accounted for most of the predictive influence, and that FLAIR- and T2-derived descriptors dominated classification behavior. These findings suggest that melanoma and NSCLC metastases may exhibit sequence-dependent phenotypic differences that can be captured through structured radiomic analysis, although reproducibility requires confirmation in larger independent cohorts.

Previous research has demonstrated the potential to predict the primary tumor origin from brain metastases. For example, Kniep et al. (2018) developed machine learning models using T1, T1CE, and FLAIR MRI data from 189 patients across multiple centers. These models incorporated radiomic features fused with clinical data to classify melanoma, lung, gastrointestinal, and breast cancers, achieving AUC scores ranging from 0.64 to 0.82. Specifically, the AUCs of 0.64 and 0.82 corresponded to NSCLC and melanoma, respectively (16). In addition, Cao et al. (2022) presented ML models that use radiomics derived from CT and MRI scans to predict the primary origin of brain metastasis. Specifically, models trained solely on the radiomic features from MRI T1CE in 78 patients achieved an AUC of 0.77 (10). The performance of the selected configurations in the present focused binary framework was within the range reported by prior studies, with test AUC values of 0.74 for RF-200, 0.75 for LGBM-220, and 0.71 for SVC-100. Importantly, direct statistical comparison across studies is not feasible due to differences in cohort composition, classification tasks, imaging protocols, and validation strategies. Therefore, the contribution of the present work lies not in outperforming prior models but in introducing a redundancy-aware framework with explicit modality-level attribution, which provides additional interpretability and insight into sequence-specific contributions. Additionally, their approach relied on multi-class classification and did not specifically isolate NSCLC as a distinct entity nor systematically quantify modality-level attribution. In contrast, the present study narrows the classification task to a clinically relevant binary comparison between melanoma and NSCLC, thereby preserving class-specific signal, structured redundancy control, and quantitative explainability through modality-level SHAP aggregation.

The prominence of FLAIR and T2 sequences in the SHAP attribution hierarchy of our framework is biologically plausible. FLAIR imaging effectively detects peritumoral edema, extracellular water, and tumor–brain interface changes (17). Melanoma brain metastases are typically T1 hyperintense due to melanin and often show susceptibility-related signal loss from hemorrhage or metal ions. In a study of 120 melanoma and 120 lung metastases, Gaviani et al. (2006) found T1 hyperintensity in 55% of melanoma lesions and T2*-weighted susceptibility loss in 42%, with both in 26% (18). Variability was observed across lesions as T1 hyperintensity was associated with melanin, but this association was not universal. Imaging characteristics are heterogeneous, with a hemorrhagic nature of melanoma metastases, reflecting variability in melanin, iron, and hemorrhage (19). This clarifies why T1 features are less significant in our analysis, which centers on sequences such as FLAIR and T2 that more effectively detect edema, heterogeneity, and hemorrhagic byproducts to differentiate melanoma from NSCLC metastases. The dominance of texture-based radiomic features further supports this view (11). Texture descriptors reflect intratumoral heterogeneity, variations in the structure around the tumor, and likely indicate differences in vascular permeability, hemorrhagic content, and inflammation among tumor types (6, 20, 21). These spatial patterns are probably more consistent across patients than isolated melanin-related T1 signal changes.

The relatively lower contribution of T1ce-derived features is notable. While contrast enhancement indicates blood–brain barrier disruption, enhancement patterns often overlap among different metastatic histologies. Ring enhancement and central necrosis occur in various primary tumors, limiting their specificity in distinguishing among them (22). In contrast, radiomic features from FLAIR and T2 sequences may detect subtle heterogeneity and microenvironmental patterns beyond visible enhancement, which could explain their greater influence in the current model (23).

Feature-level SHAP analysis indicated that classification depended on a small set of predictors that combined texture and intensity descriptors. Texture features were most influential, highlighting spatial heterogeneity and gray-level organization, which refers to the way signal intensity voxels are arranged within and around tumors, as key in distinguishing melanoma from NSCLC metastases. These descriptors focus on internal structural complexity rather than signal intensity alone. Intensity-based features also contributed meaningfully to prediction. Metrics describing distribution shape and extreme values likely reflect alterations in voxel intensity histograms associated with edema, necrosis, and haemorrhagic components, which are known to influence MRI signal characteristics in metastatic lesions (6, 21).

These findings could have important clinical implications in various practical situations. When brain metastasis is the first sign of malignancy, imaging-based characterization might help refine the differential diagnosis while awaiting results from systemic workup or biopsy. For instance, a patient with a single brain lesion and no known primary tumor could benefit from radiomic analysis, which may indicate a higher likelihood of melanoma compared to NSCLC. This could help prioritize dermatologic assessments or thoracic imaging. In multidisciplinary tumor boards, such quantitative tools can provide additional, non-invasive phenotypic insights to support management decisions, especially when imaging features are ambiguous. Furthermore, structured radiomic explanations can serve as educational tools for residents and fellows by showing which imaging patterns, such as spatial heterogeneity or edema distribution, are most influential for classification. Nevertheless, histopathologic confirmation remains the gold standard, and radiomics should be regarded as an adjunct that generates hypotheses rather than a replacement for tissue diagnosis.

Our study has several strengths. First, the use of multicenter source data and patient-level separation between training and held-out testing reduces, but does not eliminate, the risk of overfitting. Second, we applied a rigorous systematic redundancy-control methodology to interpretable features. Third, we conducted structured parametric sensitivity analyses across progressively larger top-K feature subsets, demonstrating a performance plateau beyond moderate feature counts and confirming that a compact subset captures most of the discriminative signal. In parallel, modality-level parametric analysis revealed dynamic contribution patterns across feature thresholds, providing insight into sequence-specific dominance. Lastly, multiple explainability methods have been implemented in the pipeline, including modality-specific SHAP insights, thereby enhancing interpretability.

However, the study has several limitations, including a moderate sample size and the absence of external validation, both of which may limit the generalizability of the findings. More specifically, the cohort included only 57 patients, with 16 NSCLC cases, which increases the risk of split-dependence, model instability, and optimistic performance estimation. Although broadly consistent performance trends were observed across different model families and top-K feature configurations, this consistency should be interpreted only as an internal indication that the discriminative signal was not confined to a single unstable model setup. The reported performance should therefore be considered preliminary and exploratory rather than definitive. Larger independent cohorts with adequate NSCLC representation are required to confirm the robustness and generalizability of the proposed framework. Furthermore, radiomic features are sensitive to variations in scanner vendors, acquisition protocols, and preprocessing pipelines. Although the dataset includes multicenter imaging of various magnetic fields and vendors, it does not constitute a truly independent external cohort; thus, the model’s performance may reflect dataset-specific characteristics. Finally, a direct comparison with neuroradiologists was not performed.

Future prospective reader studies are warranted to determine whether radiomics-based models provide incremental value over neuroradiologists within clinical decision-making workflows. Future research should also prioritize external validation in independent multicenter cohorts and explore integrating advanced imaging sequences, such as SWI, along with selected clinical variables to further enhance robustness and clinical applicability. Moreover, expanding the framework to encompass a broader range of tumor types and additional molecular data could yield further insights.

In summary, a redundancy-aware radiomics framework showed preliminary discrimination between melanoma and non–small cell lung cancer brain metastases using standard MRI. Feature-level attribution analysis showed that classification was driven by a concise set of texture and intensity distribution descriptors, with fluid-sensitive sequences (FLAIR and T2) playing a key role. These results indicate that spatial heterogeneity and microenvironmental signal features may offer more consistent discriminative information than contrast-enhancement patterns alone. Although histopathologic confirmation remains the gold standard, this targeted, interpretable radiomics approach may serve as a complementary, non-invasive means of elucidating the metastatic phenotype, particularly in complex or uncertain diagnoses. Further validation in larger, external cohorts is needed to verify its generalizability and to support its potential clinical value.

## Data Availability

Publicly available datasets were analyzed in this study. This data can be found here: https://www.cancerimagingarchive.net/collection/pretreat-metstobrain-masks/.
